# Early modelling of the effects and healthcare costs of the Dutch citizen-rescuer system for out-of-hospital cardiac arrests

**DOI:** 10.1371/journal.pone.0293965

**Published:** 2023-11-10

**Authors:** Anam Ahmed, Janne C. Mewes, Isabelle Lepage-Nefkens, Hanno L. Tan, Hubertus J. M. Vrijhoef, for the ESCAPE-NET Investigators

**Affiliations:** 1 Panaxea B.V., Den Bosch, The Netherlands; 2 Department of Clinical and Experimental Cardiology, Heart Center, Amsterdam Cardiovascular Sciences, Amsterdam UMC location AMC, University of Amsterdam, Amsterdam, The Netherlands; 3 Netherlands Heart Institute, Utrecht, The Netherlands; Jordan University of Science and Technology, JORDAN

## Abstract

**Objectives:**

1) to analyse the total average healthcare costs of a patient with an out-of-hospital cardiac arrest (OHCA), as well as estimating the operational costs of the citizen-rescuer system (CRS); 2) to conduct an early modelling of the effects and healthcare costs of the Dutch CRS in comparison to no CRS.

**Methods:**

A health economic modelling study was conducted. Adult patients with OHCA from cardiac causes in the province of Limburg (the Netherlands) were included. The time horizon was from OHCA occurrence up to one year after hospital discharge. First, the total average healthcare costs of OHCA patients were analysed as well as the yearly operating costs of the CRS. Second, an early modelling was conducted to compare from the healthcare perspective the healthcare costs of OHCA patients with the CRS being activated but no responders attended (CRS-NV) versus the CRS being activated with attendance of ≥1 responder(s) (CRS-V).

**Results:**

The total average healthcare costs per patient are €42,533. The yearly operating costs for the CRS are approximately €1.5 million per year in the Netherlands. The early modelling of costs and effects showed that the incremental healthcare costs per patient thus were €4,131 in the CRS-V versus the CRS-NV group (€25,184 in the CRS-V group and €21,053 in the CRS-NV group). Incremental quality-adjusted life years (QALYs) was 5 per 100 patients (16 per 100 patients in the CRS-V group versus 11 per 100 patients in the CRS-NV group). The incremental cost-effectiveness ratio (ICER) was €79,662 per QALY for the CRS-V group.

**Conclusion:**

This study shows that patients in the CSR-V group had additional health care costs of €4,131 per patient compared to patients in the CRS-NV group. This increase is caused by patients surviving more often, who then continue to utilise health services, which results in a (logic) increase in healthcare costs. For future research, accurate and up-to-date data on effectiveness and costs of the CRS needs to be collected.

## Introduction

Out-of-hospital cardiac arrest (OHCA) is one of the most common causes of mortality worldwide [1, 2]. The incidence of OHCA is expected to rise further in view of the ageing of the general population and the fact that OHCA mostly strikes elderly individuals who have accrued multiple comorbidities [3, 4]. OHCA survival rates are low with average survival rates to hospital discharge of approximately 9% in Europe [1, 5]. In the Netherlands, with a population of 17.3 million people, approximately 17,000 people suffer from an OHCA annually [6]. The chance of survival-to-hospital-discharge in the Netherlands is around 23%, higher than the European average [6, 7]. In order to improve survival chances after an OHCA, it is crucial that patients receive cardiopulmonary resuscitation (CPR) as quickly as possible, and that ventricular fibrillation (the causative cardiac arrhythmia) is terminated promptly through defibrillation, e.g., with the use of an automated external defibrillator (AED) [8–12].

In the Netherlands, when the dispatch centre is notified of a patient with suspected OHCA, two ambulances (emergency medical system, EMS) are dispatched, each staffed with a paramedic and a driver with CPR skills. Simultaneously, the police and fire brigade are alerted, as they also are trained to start CPR and perform defibrillation (if they carry an AED in their vehicle) should they arrive first. In that case they would start resuscitation and continue until the ambulances arrive; when the ambulance is to arrive first, the police and fire brigade staff assist the ambulance staff as needed.

To reduce the time to CPR and/or defibrillation, an early intervention program was set up in 2008. A citizen-rescuer system (CRS) was implemented in the Netherlands that activates volunteers who have signed up for the CRS by means of an app or text-messaging. The CRS alerts citizen-rescuers close by the victim, who generally arrive faster at the scene than the EMS and start CPR and defibrillation with an AED, thereby increasing survival chances [13]. This system consists of: 1) an AED-network and a registry-database; and 2) a network of volunteers trained in resuscitation who can be activated by app or text-message. The AED network consists of AEDs registered in a database that were acquired by municipalities and placed in strategically situated locations, or are privately-owned, e.g., by shops, residential buildings, or public facilities such as train stations. It is estimated that 25% (100,000 AEDs in total) of all AEDs in the Netherlands are currently registered. To sign up as a volunteer in the CRS, adult citizens can register after following a training course on CPR and AED use. The dispatch centre activates the CRS when they are alarmed for an OHCA suitable for attendance of volunteers. Unsuitable cases include OHCAs with suspicion of a traumatic resuscitation, OHCA victims known to have a do-not-resuscitate order or infants, OHCAs caused by suicide, or OHCAs with unclear location or locations being unreachable for the volunteer [14]. When the system is activated, the volunteers within a radius of one kilometre of the victim are notified by app or text-message. Volunteers are instructed to either directly travel to the patient to start CPR (one third of the notifications), or to pick up and bring the closest AED (two-third of the notifications) [11, 13]. The volunteers are asked to perform CPR and apply the AED (if necessary and applicable) until the EMS arrive and take over. The average time from emergency call to ambulance arrival in the Netherlands is eight minutes [7], whereas the aim is to achieve a maximum of six minutes, irrespective of the patients’ location. First evaluations of the Dutch CRS showed that citizen-rescuers on average arrive 2.5 minutes on scene earlier than EMS [15]. Studies from several European countries found the survival rate of OHCA patients to increase after the introduction of CRS, presumably through the earlier provision of CPR and defibrillation [11, 16–19].

Next to identifying the system´s effectiveness, it is important to analyse the costs and calculate the returns associated with a CRS. As data required for a full economic evaluation are still scarce, the objectives of this study were to 1) analyse the total healthcare costs of a patient with an OHCA up to one year after hospital discharge, as well as estimating the operational costs of the CRS; and 2) to conduct an early modelling of the effects and healthcare costs of the Dutch CRS in comparison to no CRS with a one-year time horizon from a healthcare perspective.

## Methods

A health economic modelling study has been conducted. First, we analysed the healthcare costs of OHCA patients up to one year after hospital discharge, as well as the yearly costs for operating the CRS. Second, we conducted an early modelling to compare the healthcare costs of OHCA patients with the CRS being activated but no responders attended (CRS-NV) versus the CRS being activated with attendance of ≥1 responder(s) (CRS-V) from the healthcare system perspective. The patient group consisted of adult patients with witnessed OHCA of cardiac origin in the province of Limburg (the Netherlands). A ’witness’ was described as an individual who observed, heard, or monitored the incident, while the term ’bystander’ was used for those individuals who were not present during the event but arrived at the location afterward.

### 1. Analysis of healthcare costs of OHCA patients and cost for operating CRS costs

The healthcare costs were analysed with a time horizon from occurrence of the OHCA up to one year after hospital discharge.

#### Data sources and input

The main data sources on costs were scientific literature [20–22], reports [23, 24], and Dutch reference prices [25]. In addition, interviews with experts (n = 5) were conducted to collect input for data that were unavailable from other resources. Interviews were held with four clinicians (i.e., cardiologists, anaesthesiologist, rehabilitation specialist), and a board member of the national organisation in charge of the CRS.

First, the healthcare costs associated with the OHCA were determined. These included costs for the EMS, hospital care (consisting of emergency room (ER) treatment, hospital stay, diagnostics, and treatment), costs incurred during the first year after hospital discharge, and end-of life costs. In Table 1 the unit costs and volumes are shown. EMS costs were based on Dutch reference prices [23, 24]. For diagnostic procedures and hospital treatment, Dutch reference prices were used if available. When unavailable, the average tariff of the pricing lists of four hospitals (two academic [26, 27] and two non-academic hospitals [28, 29]) was used. The volumes of diagnostic procedures and treatments were based on expert opinion and on the study of Van Alem et al. (2004) [22]. This study provided a detailed overview of the percentage of patients following certain procedures. However, as its data collection was conducted in 2000–2002, the experts were asked on the applicability of this data to current practice. The experts stated that in current practice the average length of stay is shorter and that the length of stay based on the Van Alem study can be reduced by 20%.

**Table 1:** Input data on costs.

**Parameter**	**Unit cost (€)**	**Volumes per patient**	**Source(s)**
EMS	784	2.0	[11, 24]
Transport EMS to hospital	31	1.0	[23, 24]
Treatment in ER	316	1.0	[23], expert opinion
Hospital stay:			
• intensive care unit	1,950	3 days	[23], expert opinion
• coronary care unit	928	3.9 days	[22], expert opinion
• cardiology ward	353	14 days	[22], expert opinion
• neurology ward	521	1.5 days	[22], expert opinion
• other wards	353	0.9 days	[22], expert opinion
Diagnostics/treatment			
**Parameter**	**Unit cost (€)**	**Volumes**	**Source**
• Cardiac catheterization	3,050	80%	[25], expert opinion
• Magnetic resonance imaging	700	20%	[25], expert opinion
• Angioplasty/ stent insertion	8,470	50%	[25], expert opinion
• Bypass surgery	14,500	10%	[25], expert opinion
• Implantable cardioverter-defibrillator	22,300	25%	[25], expert opinion
• Pacemaker	14,230	5%	[25], expert opinion
• Cardioversion	1,250	5%	[25], expert opinion
• Electrocardiogram	200	100%	[26–29], expert opinion
• Echocardiogram	550	100%	[26–29], expert opinion
• Computed tomography scan	440	40%	[26–29], expert opinion
• Electrophysiologic study	1,290	5%	[26–29], expert opinion
Healthcare costs after discharge			
• EMS costs	160	1.0	[21], expert opinion
• Hospital care	2,095	1.0	[21, 25], expert opinion
• Rehabilitation	3,260	1.0	[21], expert opinion
• Consultations (general practitioner, medical specialist, company doctor, other)	885	1.0	[21], expert opinion
• Supportive and psychological care	985	1.0	[21], expert opinion
• Medication	495	1.0	[21], expert opinion
• Special aids	2	1.0	[21], expert opinion
End-of-life costs	733	1.0	[20], expert opinion

EMS: emergency medical services, ER: emergency room.

Healthcare costs after discharge were based on Moulaert et al. [21] From this study we identified the costs applicable to the healthcare perspective. All costs were inflated to 2022 values (average exchange rate from Euros to US dollars in 2022: 1.05).

The costs of the CRS for the Netherlands were obtained during an interview with a board member of the national organisation in charge of the CRS. The costs of the CRS were considered societal costs. They were thus assessed for the purpose of placing them in perspective, but not in the early modelling below. The costs for running and maintaining the system were considered to be similar to the costs for keeping the fire brigade or ambulance services in place.

#### Analysis and outcome measure

Total average healthcare costs per patient was the main outcome measure. These were calculated by multiplying volumes of healthcare resources with the unit costs.

### 2. Early modelling of the healthcare costs and effects

An early modelling study was conducted of the effects and healthcare costs of patients with an OHCA when the CRS is activated and when at least one volunteer shows up compared to when the CRS is activated and no volunteers show up. The healthcare costs, survival, and quality adjusted life years (QALYs) per patient were analysed using a decision-tree model programmed in Microsoft Excel (Microsoft, Redmond, WA). The QALY was calculated by multiplying the 1-year survival rate after hospital discharge with the utility scores. The analysis was conducted from a healthcare system perspective over a time horizon from the OHCA to one year after hospital discharge, in the Dutch setting.

#### Model structure

**Fig 1:**
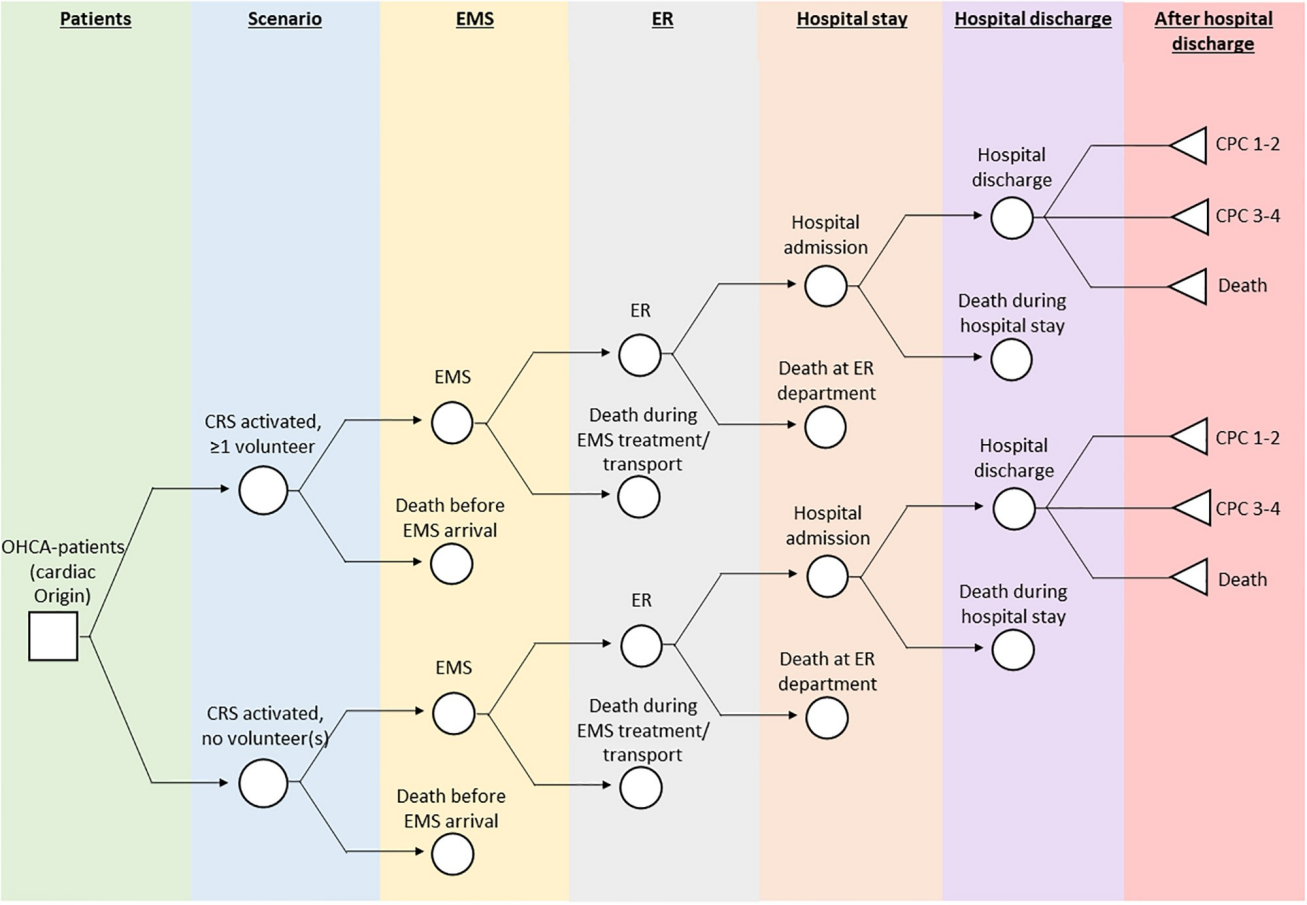
Decision tree. OHCA: out-of-hospital cardiac arrest, CRS: citizen-rescuer system, EMS: emergency medical services, ER: emergency room, CPC: cerebral performance category.

The decision-tree is presented in Fig 1. For all patients suspected of an OHCA for whom bystanders call, the emergency dispatch centre sends two ambulances and the police or fire brigade as first responders. When the CRS is in place, additionally, the volunteer network is activated when the case is suitable to be attended by volunteers. After the patient is attended by EMS staff (in the CRS-V group this is the case after volunteers attended the patient first), the patient is taken to the emergency room (ER) to receive urgent care, admitted to the hospital, and then discharged from the hospital. At all steps patients have a likelihood to die: before EMS arrival, during EMS transport or treatment, at the ER department, during hospital stay, at and after hospital discharge. When alive at hospital discharge, the patient is discharged with a Cerebral Performance Category (CPC) score of either 1–2, or of 3–4. The CPC score assesses the neurologic outcome of a patient following cardiac arrest, with a CPC score of 1–2 indicating a good cerebral performance to moderate cerebral disability; a CPC score of 3–4 indicating severe cerebral disability to the patient being in a coma/vegetative state [30].

#### Data sources and input early modelling

The main data sources were scientific literature [11, 31–35], reports [7], and interviews held with experts (n = 5). In the literature, one study [11] is available that evaluates the effect of the Dutch CRS on survival [11]. As a pre-post comparison of the implementation of the CRS was not available, the same comparison as in Pijls et al. 2016 was used: the CRS being activated but no responders attended (CRS-NV, 31% of the cases) versus the CRS being activated with attendance of ≥1 responder(s) (CRS-V, 69% of the cases). Input data on probabilities was based on the study of Pijls et al. (2016) [11]. Based on the numbers of patients surviving at departure on site, at hospital arrival and at hospital discharge in Pijls et al. (2016) [11], for our study probabilities of patients surviving were calculated for the decision-tree, see Table 2. These calculations were validated with the first author of the Pijls study. The overall survival rate to hospital discharge in the CRS-V group was 27.1%, and 16.0% in the CRS-NV group. The utility of an OHCA-patient discharged from the hospital with a CPC score of 1–2 was 0.75, and with a CPC score of 3–4 was 0.47 on a 10-points scale (Table 2).

**Table 2:** Input data on probabilities.

Parameter	CRS-V group	CRS-NV group	Source(s)
Patients alive at EMS arrival (%)	57.3	50.8	[11]
Patients alive at ER arrival (%)	99.7	99.3	[11]
Patients alive at hospital admission (%)	73.8	73.8	[31, 34]
Patients alive at hospital discharge (%)	64.3	43.0	[11]
Overall survival from OHCA to hospital discharge (%)	27.1	16	[11]
CPC 1–2 at hospital discharge (%)	92.3		[7]
CPC 3–4 at hospital discharge (%)	7.7		[7]
1-year survival after hospital discharge (%)	87.0		[32, 33]
Utility CPC-score 1–2	0.75		[35]
Utility CPC-score 3–4	0.47		[35]

EMS: emergency medical services, ER: emergency room, OHCA: out-of-hospital cardiac arrest, CPC: cerebral performance category.

#### Costs

The healthcare costs of OHCA patients up to one year after hospital discharge determined in the first objective of this study were included in the early modelling.

#### Analysis

For each pathway in the decision-tree, the probability of following that pathway was multiplied with the costs incurred along the way, leading to the total healthcare costs per patient per intervention. For the incremental costs, the cost of no CRS in place were subtracted from the CRS. Incremental effects are determined by subtracting the QALYs of the CSR-NV group from the CRS-V group.

#### Sensitivity analysis

A one-way sensitivity analysis (OWSA) was performed to assess the model results’ robustness and to identify the key drivers of the analysis. Each individual parameter was varied by +/-20% while other parameters remained at their base case value.

#### Scenario analysis

Several scenario analyses were conducted. The first two scenarios concerned increasing the survival of the CRS-V group by an absolute by 5% (scenario 1) and 10% (scenario 2) (parameters: patients alive at EMS arrival, at ER arrival, at hospital admission, at hospital discharge), as improvement of the CRS may have further reduced the time to CPR/AED since 2016. The third scenario includes the use of extracorporeal membrane oxygenation (ECMO) treatment [36] in both groups. This relatively expensive treatment is not included in the standard-of-care and is only applied to a small number of patients. In scenario three applying this treatment would increase the in-hospital costs to approximately €33,946.

#### Outcome measures

The main outcome measures were incremental costs per patient, incremental quality adjusted life-years (QALYs) per patient, and the incremental cost-effectiveness ratio (ICER).

#### Ethics

As this study did not involve patients or study participants, an ethical research approval was not needed according to Article 1b of the Dutch Medical Research in Human Subjects Act [37]. Notwithstanding, all experts were informed about the study, provided their consent, and all data were processed anonymously. The experts could withdraw from the study at any time without any consequences.

## Results

### 1. Costs OHCA-patient up to one-year after hospital discharge and of the CRS

The average healthcare costs per patient are shown in Table 3. This amount to a total of €42,533.

**Table 3:** Average healthcare costs per OHCA-patient.

Parameter	Average costs per patient (in €)
EMS	1,631
Treatment in ER	316
In-hospital costs	32,702
Healthcare costs during first year after hospital discharge	7,884

EMS: emergency medical services, ER: emergency room.

The costs for the CRS are approximately €1.5 million per year for the Netherlands. These costs consist of management of the platform; support and management of trained volunteers, AED-administrators, and local partner organizations; relationship management; functioning of the application; policy and legal duties; follow-up care for volunteers who attended a patient; and costs of communication, partner days and support (activities aimed at retaining volunteers and AED-administrators). The costs related to the recruitment of the volunteers was stated to be negligible.

### 2. Early modelling of costs and effects

#### Base case results

**Fig 2:**
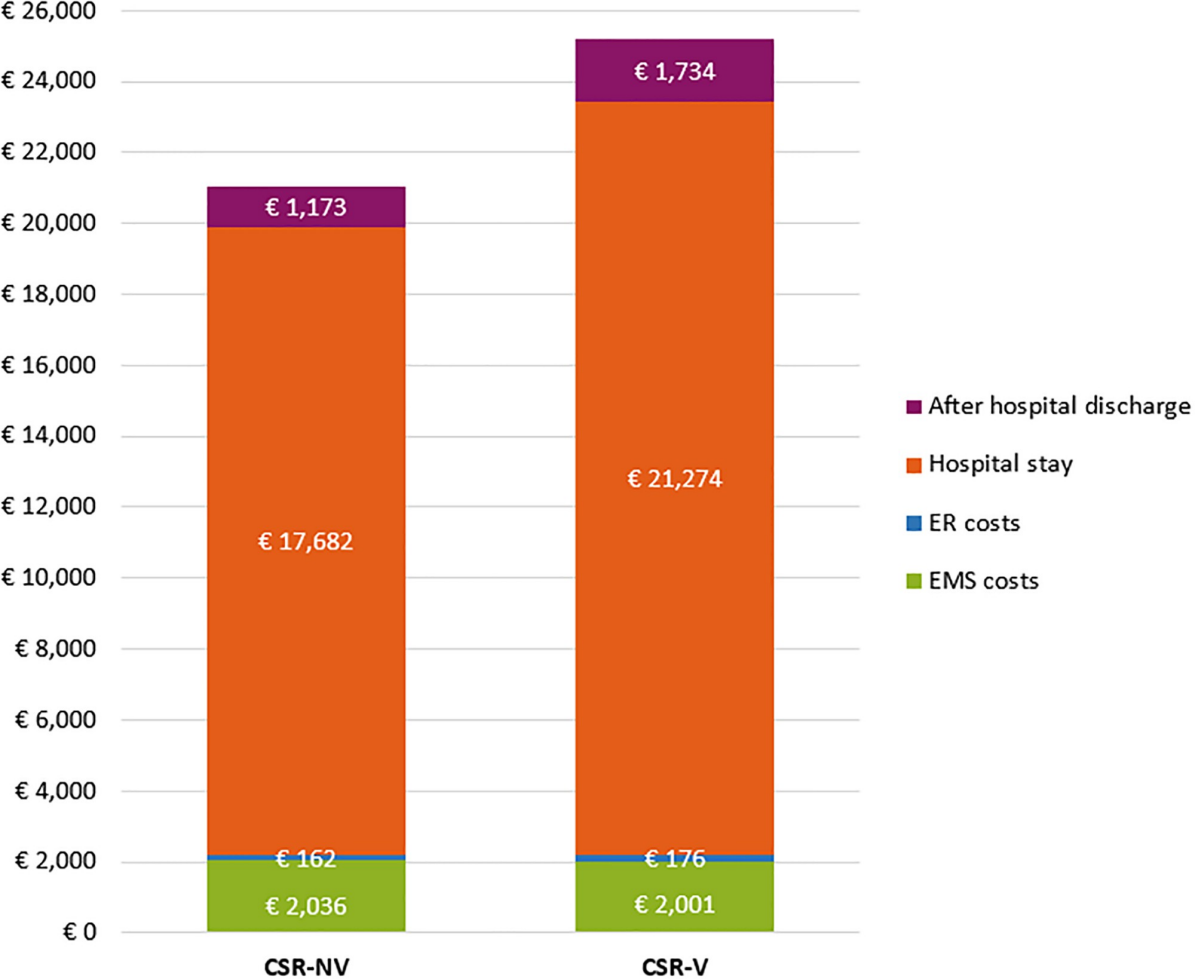
Average total healthcare costs per patient in both groups. EMS: emergency medical services, ER: emergency room, CRS-NV: citizen-rescuer system being activated but no volunteers responding to the notification, CRS-V: citizen-rescuer system being activated and at least one volunteer responding to the notification.

The average total healthcare costs per patient with an OHCA were €25,184 in the CRS-V group and €21,053 in the CRS-NV group, resulting in additional costs of €4,131 per patient in group CRS-V versus CRS-NV (Fig 2). The average number of QALYs were 16 per 100 patients in the CRS-V group and 11 per 100 patients in the CRS-NV group, resulting in an incremental QALY of 5 per 100 patients over the time horizon of one year after hospital discharge. The ICER was €79,662 per QALY for the CRS-V group.

#### Sensitivity analysis

**Fig 3:**
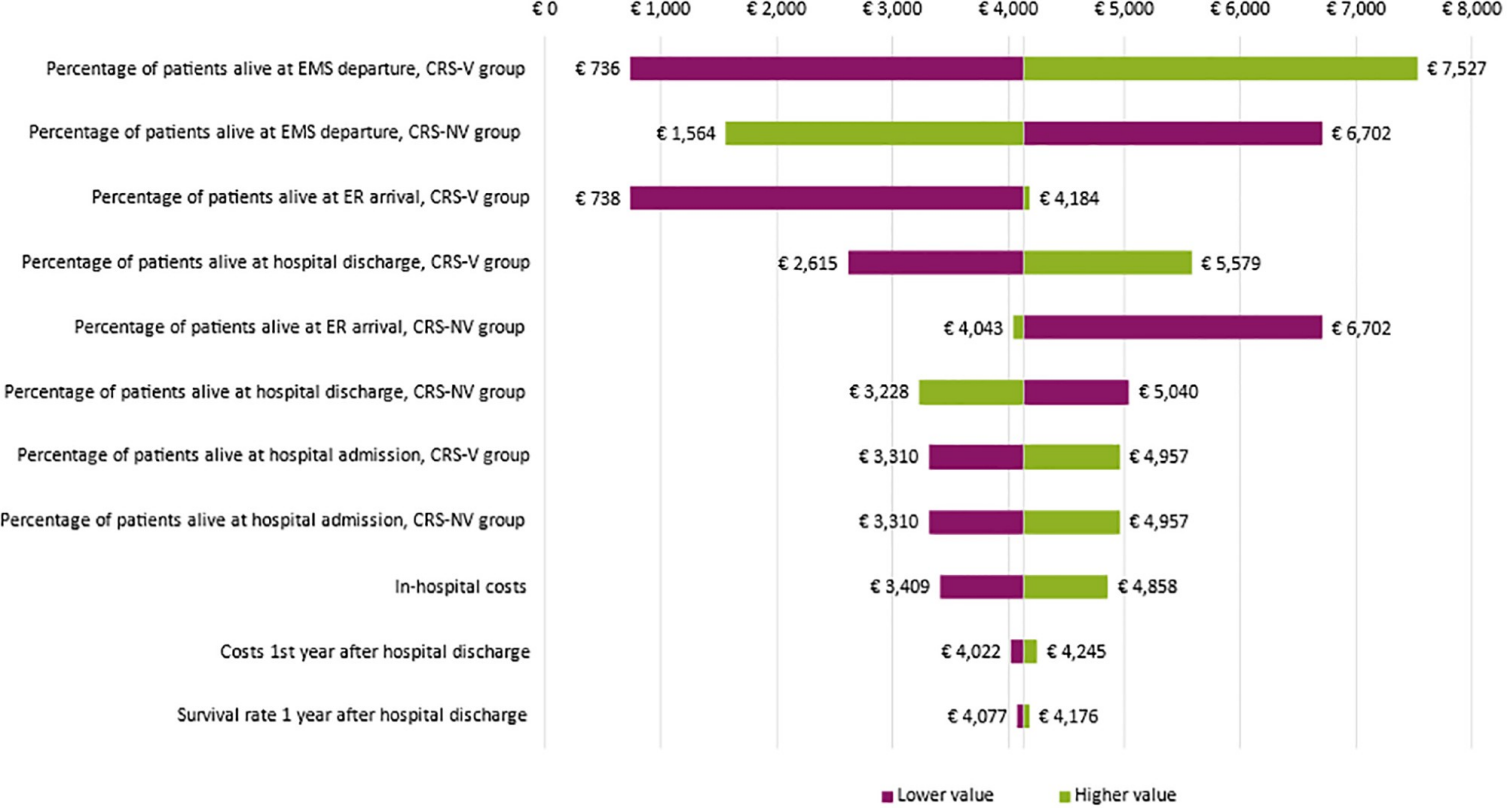
Tornado diagram sensitivity analysis.

When varying the input meters by +/- 20% one by one, the most influential parameters were: (1) percentage of patients alive at EMS departure, CRS-V group, (2) percentage of patients alive at EMS departure, CRS-NV group, (3) percentage of patients alive at ER arrival, CRS-V group, and (4) percentage of patients alive at hospital discharge, CRS-V group. The tornado diagram (Fig 3) shows the ten most influential parameters.

#### Scenario analysis

In Table 4 the outcomes of the scenario analysis are shown.

**Table 4:** Results scenario analysis.

Scenario		CRS-NV group	CRS-V group	Incremental
**Base case**	Average total costs per patient	€21,053	€25,184	€4,131
	QALYs per 100 patients	11	16	5
**Scenario 1**	Average total costs per patient	€21,053	€27,340	€6,287
**5% survival increase**	QALYs per 100 patients	11	18	7
**Scenario 2**	Average total costs per patient	€21,053	€29,546	€8,493
**10% survival increase**	QALYs per 100 patients	11	21	10
**Scenario 3**	Average total costs per patient	€21,691	€ 25,975	€4,264
**increased in-hospital costs**	QALYs per 100 patients	11	16	5

QALYs: quality adjusted life years

## Discussion

### Principal findings

In this study we analysed the average healthcare costs of a patient with an OHCA surviving up to one year after hospital discharge and conducted an early modelling of the healthcare costs and effects of the Dutch CRS with a one-year time horizon. The total average healthcare costs per OHCA-patient over the complete time horizon were €42,533. The early modelling of costs and effects showed that the incremental healthcare costs per patient thus were €4,131 in the CRS-V versus the CRS-NV group and the incremental QALY was 5 per 100 patients. The ICER was €79,662 per QALY for the CRS-V group.

The sensitivity analysis showed that the most influential input parameters were: (1) percentage of patients alive at EMS departure, CRS-V group, (2) percentage of patients alive at EMS departure, CRS-NV group, (3) percentage of patients alive at ER arrival, CRS-V group, and (4) percentage of patients alive at hospital discharge, CRS-V group. The scenario analysis demonstrated that an increase of survival in CRS-V group by an absolute 5% and 10% resulted in an incremental costs per patient of €6,287 and €8,493 respectively, and incremental QALYs of 7 per 100 patients and 10 per 100 patients respectively.

The increased costs in the CRS-V group compared to the CRS-NV group are caused by more patients surviving in that group. The treatment costs when surviving are identical per group. As in the CRS-V group more patients survive and make use of these costs, their healthcare costs increase. When a person survives or lives longer thanks to a healthcare intervention, it is very likely that this person will consume medical care during treatment and in his/her extra years of life. The inclusion of (related and unrelated) medical costs due to life years gained in economic evaluations of life-prolonging healthcare interventions remains a point of discussion [38].

### Comparison with other studies

In previous literature, Danish studies have reported on the activation of citizen responders to arrival at OHCA location and the association with bystander interventions [39–41], also during the COVID-19 pandemic [42], and have looked at variations in citizen responder availability [43]. Swedish studies found that smartphone dispatch of volunteer responders with instructions to retrieve nearby AEDs versus instructions to directly perform CPR did not statistically significant increase overall bystander AED use [44, 45]. Although there are comparable citizen responder systems in Europe, scientific literature and data on cost-effectiveness of CRSs are scarce. A cost-effectiveness analysis was conducted on the Belgian CRS [46]. The Emergency Volunteer Application is a Belgian smartphone application in which volunteers perform CPR and defibrillation with publicly available AEDs after an emergency call for suspected OHCA. In line with our study, the Belgian study demonstrated that due the smartphone application, the costs of care increased (compared to standard care), due to the increased survival of patients and also reported an increase in QALYs. The study assumed that the CPC score of patients in the intervention group generally is higher than in the control group, due to faster resuscitation of the patient, whereas in our analysis we assumed that the CPC-score of the patients who do survive until discharge for both groups are similar due to lack of evidence of the above. Therefore, this study found a higher QALY-gain than ours.

### Strengths and limitations

To the best of our knowledge, this is the first study that investigated the total healthcare costs of an OHCA up to one year after hospital discharge and the costs of the CRS and conducted an early modelling of the costs and effects of the Dutch CRS. As our study was a first and early attempt to determine the costs related to an OHCA and to model the system´s cost-effectiveness, limitations need to be considered. The main limitation of our study is the limited availability of data on the CRS. Ideally, the comparison would include either a pre-post comparison of survival rates before and after the introduction of the CRS, or a comparison of regions in which one region used the CRS and the other would not. Also, the total number of QALYs gained in relation to the incremental costs can seem rather low, however, this is caused by the short time horizon of our analysis (one year), due to the scarcity of data on long-term effects. Patients who survive up to one year after hospital discharge are expected to have a survival averaging many more years. Thus, the increased healthcare costs in the CRS-V group need to be seen as desirable, as these reflect the higher survival after the introduction of the CRS.

In this study data were based on the Pijls-study which compares getting no response from trained volunteers versus getting a response from trained volunteers when the CRS is activated. This had the disadvantage that it is not the same as having no CRS versus having a CRS. In addition, the effectiveness data of Pijls et al. (2016) [11] used in this study, may be outdated as the CRS was continually improved since 2016 (e.g., improved GPS tracking system).

### Implications for research and practice

Regarding future research, more recent data and a more extensive and long-term cost-effectiveness analysis can be conducted to capture the healthcare costs and outcomes beyond the one-year time horizon. This could involve tracking patients’ health and healthcare costs for several years post-discharge to get a comprehensive understanding of the CRS’s impact on long-term survival and quality of life. As mentioned earlier, the CRS is continually being improved since the study’s data in 2016. The impact of these ongoing improvements can be investigated, such as improved GPS tracking system, on the CRS’s effectiveness and cost-effectiveness. Moreover, challenges regarding adoption and implementation to the widespread adoption and successful implementation of the CRS in different healthcare settings can be investigated. This can help develop strategies to overcome obstacles and ensure efficient utilization. Also, the societal perspective could be adopted in the early-modelling, in which the costs of the CRS, productivity costs, costs associated with time off work for volunteers, costs related to additional care next to the intervention, and costs of the patient and the informal carers are taken into account. We had assessed the costs of the CRS system but did not include them in the modelling from the healthcare system perspective. We considered these societal costs and found it inappropriate to divide the costs among the patients experiencing an OHCA, as this is a system that was put in place as the society considers it desirable to increase survival chances after an OHCA. The costs of the CRS were analysed to be approximately €1.5 million per year. This may sound high, but must also be considered in light of costs spent on other societal provisions that may not be used by all citizens regularly, but society values these services to be in place (think e.g., of services to act on natural disaster, of which society wishes them to be available, but does not make use of them regularly or can plan when to use them). In the Netherlands, there is broad understanding and desire to have AEDs available in strategic places, such as train stations, and for the CRS. Due to the ongoing adaptions to improve the CRS, it is expected that the number of survivors with good neurological outcome will increase, implicating that the incremental QALYs increase while incremental costs will also increase. It can also be explored how the CRS affect healthcare costs and outcomes in different regions in the Netherlands. Similar systems in Europe with varying levels of healthcare infrastructure can be compared, which may help to identify contextual factors that influence the CRS’s effectiveness and cost-effectiveness. Furthermore, examining patient-centred outcomes, such as quality of life, patient satisfaction, may help understand how the CRS affects these aspects, which is crucial for comprehensive evaluation.

## Conclusion

This study showed that over the time horizon of up to one year after hospital discharge patients in the CSR-V group had additional health care costs of €4,131 per patient compared to patients in the CSR-NV group. This increase is caused by patients surviving more often, who then continue to utilise health services, which results in a (logic) increase in healthcare costs. The patients in the CRS-V group had 5 incremental QALYs per 100 patients compared to CRS-NV. The ICER was €79,662 per QALY for the CRS-V group. The additional costs for the CRS will increase as the number of OHCA survivors will also increase. For future research, accurate and real world data on effectiveness and costs of the CRS needs to be collected.

## Supporting information

S1 File Partners of the ESCAPE-NET consortium. (PDF)
