# Supplementary material for: Early modelling of the effects and healthcare costs of the Dutch citizen-rescuer system for out-of-hospital cardiac arrests
Source: PLoS One. 2023 Nov 10;18(11):e0293965. doi: 10.1371/journal.pone.0293965 (PMC10637662; doi:10.1371/journal.pone.0293965)
Supplement: S1 File — (PDF) [file pone.0293965.s001.pdf]

Supplementary file 1. Partners of the ESCAPE-NET Consortium

Region	Organisation	Members
Amsterdam, The Netherlands	Academisch Medisch Centrum (AMC)	Hanno Tan*, Marieke Blom, Irene van Valkengoed, Arthur Wilde, Aeilko Zwinderman, Elisabeth Lodder, Michael Tanck, Marcel Mannens, Laura van Dongen, Marieke Bak, Talip Eroglu, Ruud Koster, Connie Bezzina, Dick Willems, Peter Henneman, Dominic Zimmerman, Remy Stieglis, Hans van Schuppen, Michiel Hulleman, Lixia Jia, Mette Ekkel, Vera van Eeden, Robin Smits, Emma Linssen, Frank Groen
Amsterdam, The Netherlands	Stichting VUmc	Petra Elders, Sabrina Welten, Amber v/d Heijden
Den Bosch, The Netherlands	Panaxea	Anam Ahmed, Janne Mewes, Isabelle Lepage-Nefkens, Bert Vrijhoef
Copenhagen, Denmark	Region Hovedstaden	Jacob Tfelt-Hansen, Frederik Agesen, Niels Stampe, Charlotte Glinge, Simon Mathis Konig, Nertila Zylyftari, Gunnar Gislason, Fredrik Folke, Freddy Lippert, Carina Grøntved Jønck, Peder Warming
Biot, France	Societe Europeenne De Cardiologie	Gerhard Hindricks, Nikolaos Dagres, Lauren Tapp
Paris, France	Université Paris Descartes	Xavier Jouven, Jean-Philippe Empana, Marie-Cécile Perier, Eugénie Valentin
Pavia, Italy	Universita Degli Studi di Pavia	Veronica Dusi, Marta Ruffinazzi, Enrico Baldi, Gaetano M De Ferrari
Milan, Italy	Istituto Auxologico Italiano	Peter Schwartz, Lia Crotti
Milan, Italy	Istituto di ricerche farmacologiche Mario Negri	Roberto Latini, Vittorio Castiglioni, Francesca Fumagalli, Giuseppe Ristagno
Lombardy, Italy	Lombardia CARe	Simone Savastano, Enrico Baldi
Helsinki, Finland	BioComputingPlatforms (bcplatforms)	Päivi Tikka-Kleemola, Niko Hurskainen, Timo Kanninen
Stockholm, Sweden	Karolinska Institutet	Leif Svensson, Martin Jonsson, Ellinor Berglund, Andreas Claesson, Mattias Ringh, Jacob Hollenberg
Hradec Králové, Czech Republic	Zdravotnická záchranná služba Královéhradeckého kraje	Anatolij Truhlar, Monika Praunová
Brussels, Belgium	European Resuscitation Council	Bernd Walter Böttiger, Federico Semeraro, Sabine Wingen, Anja Pollnow-Schaap
Barcelona, Spain	Fundacio Istitut Mar D'Investigacions Mediques IMIM	Jordi Mestres, Andreu Bofill
München, Germany	Helmholtz Zentrum München	Thomas Metinger, Martina Kuhnert, Holger Prokisch

* Consortium leader (h.l.tan@amsterdamumc.nl)
